# Potential Host Manipulation by the Aphid Parasitoid *Aphidius avenae* to Enhance Cold Tolerance

**DOI:** 10.1371/journal.pone.0168693

**Published:** 2016-12-22

**Authors:** Lucy Alford, Annabelle Androdias, Thomas Franco, Jean-Sébastien Pierre, Françoise Burel, Joan van Baaren

**Affiliations:** UMR 6553 ECOBIO, Université de Rennes I, Avenue du Général Leclerc, Rennes Cedex, France; CSIRO, AUSTRALIA

## Abstract

During parasitoid development, the immature parasitoid is confined to the host species. As a result, any potential to modify the physiology or behaviour of the host could play an important role in parasitoid fitness. The potential for host manipulation by the aphid parasitoid *Aphidius avenae* to increase cold thermotolerance was investigated using the aphid host species *Metopolophium dirhodum* and *Sitobion avenae*. Aphids were parasitized at L3/L4 instar stage (5 d old) and allowed to develop into pre-reproductive adults (10 d old) containing a 5 d old parasitoid larva. A control group was created of non-parasitized pre-reproductive adults (10 d old). The inherent physiological thermotolerance (LT_50_) and potential behavioural thermoregulation (behaviour in a declining temperature regime) of parasitized and non-parasitized aphids were investigated. Results revealed no effect of parasitism on the physiological thermotolerance of *S*. *avenae* and *M*. *dirhodum*. Significant differences in the behaviour of parasitized and non-parasitized aphids were observed, in addition to differences between host species, and such behaviours are discussed in view of the potential for host manipulation.

## Introduction

When subjected to unfavourable thermal conditions, an organism’s survival is governed by its inherent physiological thermotolerance. For ectothermic organisms, although comparatively limited in their ability to internally thermoregulate, the inherent physiological thermotolerance that they do possess is conferred via a variety of biochemical and physiological mechanisms [1, 2]. In addition to inherent physiological thermotolerance, organisms may employ behavioural mechanisms as a form of thermoregulation, such as habitat selection across large spatial scales, involving extensive seasonal migrations to suitable overwintering sites [3], or microhabitat selection such as basking or burying [4, 5].

Whilst the role of physiological thermotolerance and behavioural thermoregulation of ectotherms is well studied, parasitic organisms and parasitoids pose an interesting scenario due to their dependence upon a host species to complete their lifecycle. Parasitoid insects, for example, which are largely dominated by the order Hymenoptera [6], lay their eggs on or within a host species and are consequently intricately linked to their host during the immature instar stage [7]. Their ability to utilise behavioural thermoregulation during immature development is subsequently severely reduced. Furthermore, should the host die prior to parasitoid pupation, so too will the developing parasitoid. As a consequence, any potential to modify the behaviour or physiology of the host could play an important role in parasite and parasitoid fitness [8].

Changes in physiology or behaviour following parasitization by a parasitoid wasp (or infection by a parasite more generally) may evolve if the change results in greater fitness, either to the parasite or to the host [9, 10]. Indeed, the majority of documented changes to parasitized animals are behaviours, considered parasite adaptations, acting to enhance transmission success [9]. Host adaptations are also plausible whereby changes may enhance host fitness, for example by aiding parasite removal or compensating against the effects of parasitization [9, 11]. However, to be truly considered an adaptive manipulation and not simply a pathological by-product of infection, the change must result in an increase in fitness for either the parasite or the host; a variable that is often difficult to measure [9].

Within the context of parasitoid wasps, documented changes to the host following parasitization fall into the category of parasite adaptations, with enhanced parasitoid fitness commonly attributed to behavioural changes induced in the host which bring about a reduction in the rate of predation and hyperparasitism [12–14], However, whilst host manipulation by parasitoids is well documented, the potential for host manipulation to enhance thermotolerance is a topic that has received considerably little research attention. There is evidence to suggest that a parasitoid may alter both the physiology and the behaviour of the host to withstand or avoid unfavourable thermal conditions, although both mechanisms have been studied in isolation.

Physiological changes to the host following parasitization within the context of thermal tolerance have received little research attention [15, 16] and have not been studied for the aphid parasitoids. However, comparison of two separate studies suggests that physiological modification acting to aid thermotolerance may occur in the Russian wheat aphid, *Diuraphis noxia*, when parasitized by *Aphelinus albipodus*, *Aphelinus asychis* and *Diaeretiella rapae*. Here, non-parasitized *D*. *noxia* possessed a supercooling point of -25°C [17]. However, the supercooling point is lowered to -30° and below when parasitized [18], suggesting that parasitism may enhance the cold tolerance of the host via physiological changes. Since the parasitoid is intricately linked to its aphid host, premature death of the aphid (i.e. prior to parasitoid pupation) will result in the death of the parasitoid also. Therefore, any ability to enhance the thermotolerance of the aphid host, thus enhancing aphid survival at unfavourable temperatures, would concurrently act to increase parasitoid survival and thus fitness.

In contrast, the potential for behavioural host manipulation by parasitoids has received much research attention since the early work of Brodeur and colleagues [12, 13, 19]. Host manipulation by parasitoids resulting in a beneficial change in host behaviour has now been documented in *Drosophila* species (*D*. *melanogaster* and *D*. *subobscura*) parasitized by *Asobara* species [20, 21] and aphids including the pea aphid (*Acyrthosiphon pisum*) and the potato aphid (*Macrosiphum euphorbiae*) parasitized by *Aphidius* species [12, 19, 22]. Here, behavioural host manipulation to aid thermotolerance has been attributed to the occurrence of alternative pupation sites by parasitized and non-parasitized insects. For insects, the choice of pupation site carries important survival consequences since during this immobile stage of development, insects are vulnerable to the hazards of desiccation, climatic conditions, fungal infection, predation and parasitism, and hyperparasitism [20]. There therefore exists strong selection to optimize the timing and location of pupation to reduce the potential risks [20]. As such, any ability of the parasite to modify pupation site of the host which reduces such risks and aids survival shall be favoured by selection. In aphids parasitized by *Aphidius ervi*, mummification occurred on the adaxial leaf surface in the upper plant canopy. Here, temperatures are approximately 2°C higher than the preferred microhabitat of non-parasitized aphids: the mid-canopy [22]. Since this new habitat is closer to the optimum development temperature of the parasitoid, this observation is suggestive of behavioural host manipulation by the parasitoid to ensure pupation occurs under more favourable thermal conditions [22]. A similar behaviour was reported for aphids of *M*. *euphorbiae* parasitized by *Aphidius nigripes*, whereby aphids parasitized by non-diapausing parasitoids mummified in the upper canopy; believed to provide a passive thermoregulation advantage to the developing parasitoid [19]. In contrast, aphids parasitized by diapausing parasitoids mummified in concealed microhabitats, offering protection against low temperatures [12]. Variation in pupation site may further act to reduce the risk of hyperparasitism [12, 19]. For this reason, elucidating the exact driving force behind these behavioural modifications is complicated and such behaviours could be the result of multiple selection pressures.

Whilst much research has focused on the effect of parasitism on site of pupation, little is known about the effect of parasitism on behaviours attributed to behavioural thermoregulation. Aphids have been shown to engage in dropping-behaviour in response to unfavourable temperatures [23, 24]. It is believed that this dropping-behaviour, also observed as an escape mechanism against predatory attack [25, 26], enables the aphid to quickly escape to more thermally suitable environments, thus avoiding thermal stress and enhancing survival [23]. Since the majority of adaptive changes following parasitization involve simple increases or decreases in an activity already performed prior to infection [9], this dropping behaviour displayed by aphids could provide a pre-existing behaviour on which host manipulation could act.

The current paper aims to provide the first study to investigate the potential for manipulation of both physiological and behavioural aspects of cold tolerance. Cold tolerance was chosen, since cold stress represents an important selective pressure acting on aphid parasitoids in North-western France [27]. The focus species for the study include the aphid parasitoid *Aphidius avenae* Haliday (Hymenoptera: Aphidiinae) and two of its cereal aphid prey, *Metopolophium dirhodum* Walker and *Sitobion avenae* Fabricius (Homoptera: Aphididae). Two aphid species were selected for experiments because aphid parasitoids are capable of utilising multiple aphid species as hosts. These two aphid species are already known to differ in their inherent thermal tolerance capacities, with *S*. *avenae* being significantly more cold tolerant than *M*. *dirhodum* [24]. Furthermore, aphid hosts are known to impact the morphology and some life history traits of the emerging parasitoid [28, 29]. As such, an effect of host species cannot be ruled out and may impact the intensity and / or the form of host manipulation observed, particularly if host species differ in their inherent thermal tolerance. By comparing parasitized and non-parasitized aphids, the current study investigates the potential for the aphid parasitoid *A*. *avenae* to manipulate the physiological thermotolerance (as determined by measurement of the LT_50_) and behavioural thermoregulation (as determined by studying behaviour in a declining temperature regime) of two species of its cereal aphid hosts, *S*. *avenae* and *M*. *dirhodum*.

The following hypotheses are tested: 1) Parasitized aphids display lower values of LT_50_ (i.e are more cold tolerant) than non-parasitized aphids as a result of physiological host manipulation, 2) Physiological thermotolerance will differ between aphid species, regardless of parasitized status, due to differences in their thermal tolerance capacity, with *S*. *avenae* being more cold tolerant [24]. This in turn will impact the intensity and / or the form of host manipulation observed for each species, with a greater degree of manipulation required for less thermal tolerance hosts i.e. *M*. *dirhodum* 3) Parasitized and non-parasitized aphids will display different behaviours at low temperatures as a result of behavioural host manipulation, with parasitized aphids displaying behaviours beneficial to low temperature survival e.g. increased dropping behaviour [23].

## Materials and Methods

### Aphid collection and rearing

Stock cultures of anholocyclic *Sitobion avenae* and *Metopolophium dirhodum* were established using aphids originally collected between March and May 2014 in the LTER site Armorique (48° 36 'N, 1° 32' W). Permission was obtained from local landowners prior to aphid collection and field work did not involve endangered or protected species. Over 50 aphids of each species were originally collected from the field and an initial quarantine period was carried out to ensure that the aphids were not host to parasitoid wasps before aphids were added to the culture. Aphids were reared on winter wheat, *Triticum aestivum*, ‘Renan’ cultivar grown in vermiculite within Plexiglas cages (50 x 50 x 50 cm) and housed in a controlled environmental room at 20±1°C and LD 16:8 h photoperiod.

### Parasitoid collection and rearing

Aphid mummies (dead aphids containing a developing parasitic wasp pupa) were collected in wheat, triticale and clover fields near Rennes (Brittany, France) in October and November 2014, approximately 3 to 4 months prior to use in experiments. Resultant parasitoids to emerge from the mummies where first identified to the species level and individuals of *Aphidius avenae* retained and used to establish a laboratory culture. A minimum of 50 parasitoids were used to establish the culture, with additional mummy collection occurring throughout December and January to maintain genetic diversity of the laboratory culture. The *A*. *avenae* culture was maintained within Plexiglas cages (50 x 50 x 50 cm) at 20±1°C and L16:D8 on the aphid *S*. *avenae* and fed on a solution of honey and water. Pots of winter wheat infested with *S*. *avenae* were added to the cages containing the parasitoid wasps three times a week to provide the wasps with a continuous supply of aphid hosts.

### Obtaining parasitized and non-parasitized aphids for experiments

To obtain aphids parasitized by *A*. *avenae*, aphid nymphs were selected from aphid stock cultures and examined under an optical microscope. Individual nymphs were selected of the L3 and L4 nymphal stage (approximately 5d old at 20°C), which represents the preferred instar for the *Aphidius* parasitoids [30–32]. Using a fine paintbrush, ten L3/L4 nymphs were placed simultaneously in a Petri dish containing a mated female of *A*. *avenae*. The Petri dish was observed by eye until the female *A*. *avenae* had oviposited within an aphid. The parasitism success rate of *Aphidius* species has been shown to be greater than 95% [33]. The newly parasitized aphid was subsequently removed from the Petri dish and a new aphid nymph placed inside. Aphid density within the Petri dish was maintained at approximately ten to ensure a relatively high encounter rate between the parasitoid and the aphid prey. The procedure was repeated until the required number of parasitized aphids for the experiment in question was obtained. During the course of the experiment, over 20 different female wasps were used to obtain parasitized aphids with each female parasitizing no more than 20 aphids. Newly parasitized nymphs were placed within a microcage (L = 16 cm, Ø = 4 cm) comprised of winter wheat grown in vermiculite at densities of 15–20 nymphs per cage and allowed to continue development for 5 days at 20±1°C and L16:D8. At 10d old (pre-reproductive adult stage), the aphids, each containing a 5d old parasitoid larva, were used for experiments. A total of 545 *S*. *avenae* (283 parasitized) and 568 *M*. *dirhodum* (234 parasitized) were used to complete the experiments detailed below.

Experiments were also performed on a control group of non-parasitized individuals. To obtain non-parasitized aphids, the procedure detailed above was repeated with the exception that the aphid nymphs were not parasitized by *A*. *avenae*. Aphids were taken from the stock culture at the L3/L4 stage, transferred to microcages and kept at the same conditions as the parasitized aphids before use in experiments at 10d old.

To minimise potential maternal effects, caused due to a telescoping of generations exhibited by aphids [34], treatment and control aphids were reared within microcages containing young wheat blades of similar age (approximately 4 days since sowing) and density. Microcages were subsequently kept within the temperature controlled chamber and thus at identical conditions to stock culture aphids.

### Determination of lower lethal temperature (LT_50_)

When measuring inherent physiological thermotolerance, a measure commonly employed in the literature is that of the lethal temperature 50 (LT_50_); that is, the temperature at which an experimental population experiences 50% mortality [24, 35–39]. The LT_50_ offers an easy to measure reference point of thermotolerance, enabling rapid comparison between treatment groups.

LT_50_ was determined for apterous pre-reproductive adults of *M*. *dirhodum* and *S*. *avenae* obtained using the method detailed above. A ‘direct plunge’ protocol was employed [40, 41]. For this, test aphids were directly exposed to a single low temperature for a duration of 2 h. This was repeated with multiple test aphids exposed to a series of sub-zero temperatures (in the region 0 to -12°C at 1°C intervals) to encompass a range of temperatures which resulted in approximately 100% aphid survival at the high temperature end, through to 0% survival at the low temperature end. Due to inter-species variation in thermal tolerance, the range of exposure temperatures was tailored to each species to incorporate 0 to 100% mortality. For this reason, the number of test insects required to calculate an LT_50_ value for each species was not identical. For each exposure temperature, 30 parasitized and 30 non-parasitized aphids were placed within 0.5ml Eppendorf tubes at densities of ten individuals per tube. The Eppendorf tubes were then placed individually within a glass boiling tube and the boiling tubes sealed with a sponge stopper to limit air circulation and maintain a stable internal environment. The boiling tubes were held within a test tube holder and lowered into the alcohol bath (Haake F3, Thermo Electron Corp., Karlsruhe, Baden-Württemberg, Germany) set to the desired sub-zero temperature. A thermocouple was placed within an empty Eppendorf tube, set up in the same way as the tubes containing the aphids, enabling the accurate monitoring of the temperature that the aphids experienced.

Following the 2 h exposure period, test aphids were transferred to recovery tubes to recover at the culture temperature of 20°C. Recovery tubes were constructed from blades of *T*. *aestivum* placed within small glass boiling tubes (Ø = 10 mm). Cotton wool soaked in water was placed in the bottom of each tube to keep the *T*. *aestivum* fresh for the duration of aphid recovery. The cotton wool was then covered in a layer of fine sand to prevent aphids coming into contact with the water, and the tubes sealed with fine netting. Survival was assessed 48 h after exposure. At 20°C, parasitoids pupate within the aphid host, forming an aphid mummy, at approximately 9–10 days following parasitization. Assessing survival after 48 h therefore resulted in individual aphids of the parasitized treatment containing a 7 d old parasitoid larva and ensuring pupation did not occur during the duration of the experiment. The procedure was repeated for each exposure temperature. A handling control was set up on each day of experiments, as detailed above, with the exception that the aphids remained at 20°C for the duration of the exposure period. The experiment was repeated for both parasitized and non-parasitized *M*. *dirhodum* and *S*. *avenae*.

### Determination of aphid behaviour at extreme low temperatures

Aphid behaviour in a declining temperature regime was measured for apterous pre-reproductive adults of *M*. *dirhodum* and *S*. *avenae* using a glass column connected to a programmable alcohol bath [24] to elucidate variation in aphid behaviour between parasitized and non-parasitized aphids in response to a sudden cold stress.

To obtain host plant material, individual grains of winter wheat were sown in plastic cylinders (L = 9 cm, Ø = 2.5 cm in diameter) containing moist vermiculite. The diameter of the plastic cylinders was chosen because it allowed the cylinders containing the wheat blade to be inserted into the glass column, thus enabling the use of live plant material in experiments as opposed to excised material. Wheat was selected for experiments following 6–9 days of growth at 20°C when the wheat blades measured approximately 7cm in height.

In all experiments, 5 new aphids (i.e. not aphids previously used in the determination of LT_50_) were transferred onto the adaxial surface of a single wheat blade using a fine paintbrush. The aphids were allowed to settle onto the wheat blade during a 30 min acclimatization period. The plastic cylinder containing the single wheat blade and test aphids was subsequently inserted into the bottom of the glass column pre-set to the culture temperature of 20°C and the glass column sealed with a sponge stopper to reduce air flow and maintain a stable thermal environment within the inner column. The programmable alcohol bath was set to decrease the temperature of the column from 20°C to -10°C at a rate of 0.75°C min^-1^. The rate of 0.75°C min^-1^ was chosen to prevent inducing a rapid cold hardening response in the test aphids [42, 43]. Furthermore, the relatively short duration time of the experiment acted to increase the likelihood of aphid movement being the direct consequence of the stress exposure and not spontaneous movement. This is because aphids feed almost continuously on the host plant [44] and will move on or from the plant only in the case of stress e.g. temperature stress [23], predation threat [25, 45] and plant senescence [46].

The main behaviours exhibited by aphids when exposed to the declining temperature regime were categorised in accordance with the behaviours detailed in Alford et al. [24]. Three main categories of behaviour were established including: 1) the aphid actively walked from the wheat; 2) the aphid dropped from the wheat; 3) the aphid remained attached to the wheat until -10°C was reached. -10°C was chosen because, at this temperature, aphids have long ceased movement and have entered a state of chill coma (a temperature induced torpor), and, as such, no additional movement from the wheat is observed [24]. During the experimental cooling regime, behavioural responses were scored for each aphid as one of the pre-established categories of behaviour, along with the current temperature within the glass column. Temperature was recorded manually from the thermocouple display reading to an accuracy of 0.1°C. The experiment was repeated with the treatment group of parasitized *M*. *dirhodum* and *S*. *avenae* and the control group of non-parasitized aphids to obtain results for 30–40 individuals of each species x treatment combination.

### Statistical analysis

The temperature resulting in 50% mortality of the experimental populations at low temperature exposures (the LT_50_) was determined using Probit analysis in MINITAB, version 17 (Minitab Inc., State College, Pennsylvania). Handling controls resulted in 99–100% survival across all treatments. The natural response rate was therefore assumed to be close to zero and not included in the model. Significant differences in mortality were identified by non-overlapping 95% fiducial limits [24, 47]. To analyse aphid behaviour in a declining temperature regime, categorical data were analysed using a chi-square test in MINITAB version 17 to determine if the proportion of individuals exhibiting each behaviour type differed between the treatment groups and species. Of the aphids which dropped from the wheat, the temperature at which this occurred was analysed to determine any effect of parasitism on this behaviour. Distribution fitting analysis was performed for each species x treatment group to determine which distribution best described the data. The normal distribution was found to provide the best overall fit for the data. Parametric distribution analysis was subsequently performed using normal as the appropriate distribution to allow for comparison of scale and location parameters. Individual comparisons were made using Bonferroni 95% confidence intervals [48–50].

## Results

### Lethal temperature

**Fig 1:**
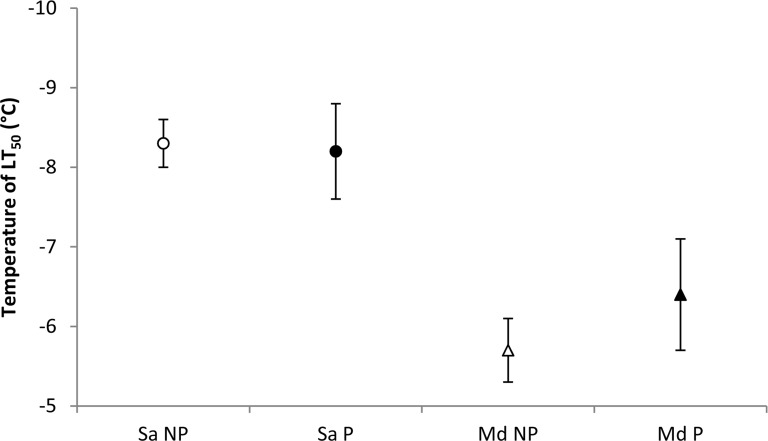
Lower lethal temperatures (LT_50_) ± 95% fiducial limit (°C) of non-parasitized adults and adults parasitized by *Aphidius avenae* of the aphids *Sitobion avenae* (indicated by circle symbols) and *Metopolophium dirhodum* (indicated by triangle symbols) acclimated at 20°C. Parasitized individuals are represented by filled symbols. (Notations are as follows: Sa = *Sitobion avenae*, Md = *Metopolophium dirhodum*, P = aphids parasitized by *Aphidius avenae*, NP = non-parasitized aphids) (Sa-NP n = 220; Sa-P n = 237; Md-NP n = 294; Md-P n = 201).

LT_50_ did not significantly differ between non-parasitized individuals and individuals parasitized by *A*. *avenae* of the cereal aphid species, *M*. *dirhodum* and *S*. *avenae* (Fig 1). The LT_50_ of *S*. *avenae* was significantly lower than *M*. *dirhodum*, irrespective of treatment.

### Aphid behaviour at extreme low temperatures

**Fig 2:**
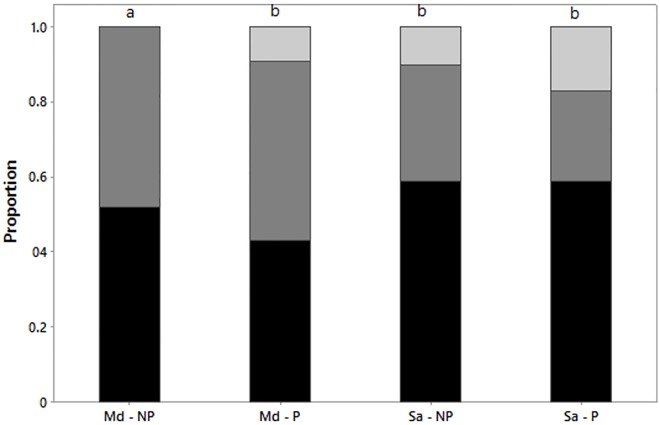
The relative proportion of behaviours exhibited by non-parasitized adults and adults parasitized by *Aphidius avenae* of the aphids *Sitobion avenae* and *Metopolophium dirhodum* when subjected to a declining temperature regime from 20°C to -10°C at 0.75°C min^-1^. Behaviours include: 1) the aphid actively walked from the wheat (light grey); 2) the aphid dropped from the wheat (dark grey); 3) the aphid remained attached to the wheat to temperatures of -10°C (black). Significant differences are indicated by letter superscripts. (Notations are as follows: Sa = *Sitobion avenae*, Md = *Metopolophium dirhodum*, P = aphids parasitized by *Aphidius avenae*, NP = non-parasitized aphids) (Md-NP n = 40; Md-P n = 33; Sa-NP n = 42; Sa-P n = 46).

The behaviours exhibited by parasitized *M*. *dirhodum* during low temperature exposure proved significantly different from the remaining treatment groups (χ^2^_2_ = 6.693, p = 0.035), with significantly more parasitized *M*. *dirhodum* actively walking from the wheat during low temperature exposure (9% compared to 0%) (Fig 2). For this group, 0 aphids actively walked from the wheat, 19 aphids dropped from the wheat, and 21 aphids remained attached to the wheat out of a total of 40. There was no significant difference in the behaviours exhibited by parasitized *S*. *avenae*, non-parasitized *S*. *avenae* and parasitized *M*. *dirhodum* during low temperature exposure (χ^2^_4_ = 6.317, p = 0.177) (Fig 2). For this group of non-significance, 15 aphids actively walked from the wheat, 40 aphids dropped from the wheat, and 66 aphids remained attached to the wheat out of a total of 121. Although more parasitized *S*. *avenae* individuals actively walked from the wheat during low temperature exposure than non-parasitized individuals (17% compared to 10%), this difference did not prove significant (Fig 2).

**Fig 3:**
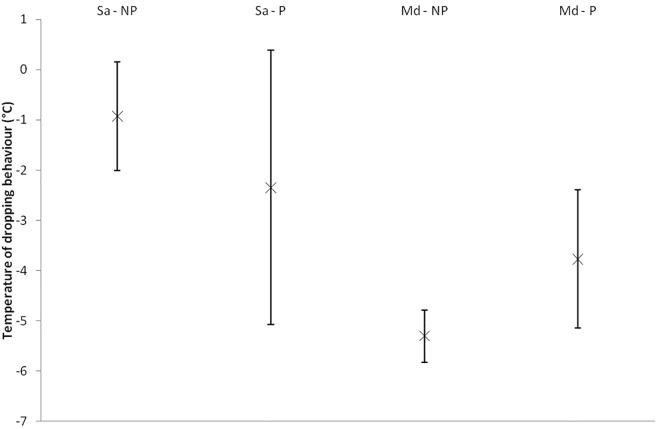
Mean temperature of dropping behaviour (°C) ± 95% confidence intervals for non-parasitized adults and adults parasitized by *Aphidius avenae* of the aphids *Sitobion avenae* and *Metopolophium dirhodum* acclimated at 20°C. These aphids represent the same test individuals as indicated by the dark grey bar in Fig 1. (Notations are as follows: Sa = *Sitobion avenae*, Md = *Metopolophium dirhodum*, P = aphids parasitized by *Aphidius avenae*, NP = non-parasitized aphids) (Sa-NP *n* = 13; Sa-P *n* = 11; Md-NP *n* = 19; Md-P *n* = 16).

Of the aphids which dropped from the wheat, there was no significant effect of parasitism on the temperature at which this occurred for *S*. *avenae*, as indicated by the location parameters (χ^2^_1_ = 0.90, p = 0.343) (Fig 3). However, for *M*. *dirhodum*, there was a significant effect of parasitism on the temperature of dropping behaviour (χ^2^_1_ = 4.20, p = 0.040), with parasitized individuals dropping from the wheat at significantly higher temperatures than non-parasitized individuals (mean temperature of dropping ± 95% CI of -3.8 ± 1.4°C and -5.3 ± 0.5°C respectively) (Fig 3). In addition, there was a significant effect of parasitism on the spread of dropping behaviour data, as indicated by the scale parameters, for both *S*. *avenae* (χ^2^_1_ = 8.46, p = 0.004) and *M*. *dirhodum* (χ^2^_1_ = 14.05, p < 0.001). For both species, the spread of data for parasitized individuals was significantly greater than for non-parasitized individuals, indicating that non-parasitized individuals dropped from the host plant over a more compressed temperature range (Fig 3).

## Discussion

### Manipulation of physiological thermotolerance

Due to the parasitoid being confined to an aphid host during development, any ability to modify the behaviour or physiology of the host to aid survival could be vital to parasitoid fitness [8]. The current study found that the temperature of LT_50_ did not significantly differ between non-parasitized and parasitized individuals of *S*. *avenae* and *M*. *dirhodum*, suggesting that the aphid parasitoid *A*. *avenae* is unable to modify the aphid’s physiology to aid thermotolerance and thus not consistent with hypothesis 1. Behavioural host manipulation is well documented in the literature [12, 13, 19–22], with several publications already on the manipulation of aphid behaviour by their parasitoids. Conversely, the ability to manipulate physiological aspects of thermal tolerance has rarely been demonstrated. Behavioural mechanisms may thus represent the most parsimonious route by which host manipulation may occur. Nonetheless, comparison of work performed by Butts [17] and Nowierski and Fitzgerald [18] suggests a change in physiological thermotolerance following the parasitization of *D*. *noxia* and, as such, both behavioural and physiological aspects should be studied to provide a complete investigation into the mechanisms of host manipulation. It is further plausible that the ability to manipulate the thermal physiology of the host may be dependent upon the ability of the parasitoid to manipulate the host. That is, the parasitoid used in the current study, *Aphidius avenae*, may be unable to significantly manipulate the host’s thermal physiology, whilst *Aphelinus albipodus*, *Aphelinus asychis* and *Diaeretiella rapae* can [17, 18]. Furthermore, it is also possible that aphid hosts may differ in their susceptibility to physiological manipulation.

Whilst no effect of parasitism on physiological thermotolerance was observed, *S*. *avenae* was consistently more cold tolerant than *M*. *dirhodum*, irrespective of treatment, thus supporting hypothesis 2. This confirms previous research that found adult *S*. *avenae* to be significantly more cold tolerant than *M*. *dirhodum* [24]. Furthermore, this species difference in cold tolerance is maintained following parasitism.

### Manipulation of behavioural thermoregulation

Although results suggest that *A*. *avenae* is unable to manipulate physiological thermotolerance, effects of parasitism on aphid behaviour were observed, offering support to hypothesis 3, with differences according to the host species. For *M*. *dirhodum*, significantly more parasitized individuals than expected walked from the wheat when exposed to extreme low temperatures than non-parasitized individuals. Although more parasitized *S*. *avenae* walked from the wheat, this difference in behaviour proved non-significant. It is possible that the observed behaviour of parasitized *M*. *dirhodum* actively leaving the wheat could represent a form of behavioural thermoregulation, with the aphid, containing the parasitoid larva, seeking out more favourable microhabitat in response to the sudden and unexpected change of temperature. Fine-scale migration by aphids to avoid unfavourable conditions has been observed in *Myzus persicae* in response to host plant senescence, with aphids migrating to younger leaves of higher nutritional value [46]. It is plausible that fine-scale migration may also be employed to seek out microhabitat of more favourable thermal conditions. Altered microhabitat choice post-parasitization could therefore act to enhance parasitoid fitness by reducing detrimental cold exposure, and, as such, may be considered a parasite adaptive change in behaviour.

In line with the host suicide hypothesis [51], an alternative explanation for the observed behaviour is that there is no host manipulation. Instead, parasitized aphids move away from the colony in order to protect the colony, comprised of closely related kin, from potential future parasitism. Using the pea aphid, *Acyrthosiphon pisum*, and the parasitoid *Aphidius ervi* as the study system, McAllister and Roitberg [51] found that parasitized aphids exhibited suicidal behaviour by enhancing their probability of dying to the benefit of the aphid colony. The behaviour of moving away from the colony could thus prove adaptive if it acts to reduce future parasitism rates of the colony. This would be particularly true in aphid species exhibiting an anholocyclic lifecycle in which parthenogenetic reproduction occurs since aphids in close proximity are likely to be genetically identical clones and, as such, the fitness benefits of suicidal behaviour would be great. Such a change in behaviour would thus represent a host adaptive change as opposed to a parasite adaptive change (i.e. host manipulation), acting to minimise the effect of parasitism on the genetic clone [9, 11].

Of the aphids that dropped from the wheat, parasitism by *A*. *avenae* did not impact the relative frequency of individuals exhibiting the behaviour. However, parasitism did negatively impact the temperature at which this occurred for *M*. *dirhodum*. Parasitism significantly raised the temperature of dropping behaviour of parasitized *M*. *dirhodum*. For *S*. *avenae*, parasitism did not significantly impact the temperature at which dropping behaviour occurred. Recent work by Ma and Ma [23] suggests that aphids employ dropping behaviour to avoid unfavourable high temperatures, in a similar way in which dropping behaviour is employed to escape from predators [25, 26; 45]. Since parasitized *M*. *dirhodum* dropped from the plant at warmer sub-zero temperatures than non-parasitized individuals, this could provide evidence that aphids may also employ dropping behaviour to avoid unfavourable low temperatures.

Results further revealed parasitism to increase the temperature range over which individuals dropped from the wheat for both species. This result could suggest a detrimental impact of parasitism on the normal functioning and physiology of the aphid host, either as a result of the direct trauma of being parasitized, biochemical changes, or resource utilisation by the parasitoid, with the impacts of parasitism on normal host development and functioning well documented in the literature [52–54]. Alternatively, this increased range over which parasitized aphids fall from the wheat could represent intraspecific variation in the ability of the parasitoid to manipulate the host or the host to resist manipulation.

It is interesting to note that significant differences in the behaviour of parasitized and non-parasitized individuals were observed for *M*. *dirhodum* only. Whilst we cannot rule out the possibility that observed differences result from variation in life history of the two species, inter-species differences could indicate variation in the susceptibility of the species to host manipulation, offering support to hypothesis 2. However, *S*. *avenae* exhibits a significantly higher level of cold tolerance than *M*. *dirhodum* and displays a high propensity to actively walk from a plant at unfavourable low temperatures [24]. As such, there may be less selection pressure acting on the ability to manipulate host cold tolerance, since the inherent physiological thermotolerance of the aphid is great enough to permit survival at the temperatures commonly experienced in the cereal fields of temperate Europe. This theory requires further testing and could form the basis for future research.

The current study therefore highlights changes to aphid behaviour following parasitization by the aphid parasitoid wasp *A*. *avenae*, further revealing variation in the degree of post-infection change between host aphid species. Since the majority of adaptive changes following infection by a parasite are attributed to increasing or decreasing the frequency of an already pre-existing behaviour [9], the increased propensity of parasitized *M*. *dirhodum* to walk from the plant at unfavourable temperatures could represent an ability of *A*. *avenae* to subtly manipulate a pre-existing aphid behaviour to avoid temperature stress. However, when interpreting such results, Poulin [9, 10] warns that care must be taken since observed changes may simply be a pathological side-effect of parasite infection. This is particularly true in cases where the observed change appears to benefit neither the host nor the parasite [9]. In the current study, the experimental set-up did not enable the measurement of resultant parasitoid fitness and, as such, we cannot conclude with certainty if the observed changes to aphid behaviour are beneficial to the parsitoid. However, since there is much research evidence to suggest that manipulation by parasitoids impacts the mobility of the hosts [12, 14, 19–22], it is possible that the change in behaviour observed in the current study is also favourable to the parasitoid host and, if shown to enhance parasitoid fitness, may be classified as host manipulation and not simply a pathological by-product.

### Conclusion

Parasitism by *A*. *avenae* did not affect aphid physiological thermotolerance, as measured by LT_50_. However, a significant difference between the behaviour of parasitized and non-parasitized *M*. *dirhodum* was observed, with parasitized *M*. *dirhodum* displaying a higher propensity to actively walk from the wheat following exposure to low temperatures than non-parasitized *M*. *dirhodum*. In addition, parasitized *M*. *dirhodum* dropped from the wheat at significantly warmer temperatures than non-parasitized individuals. Such behaviours could suggest an ability of the parasitoid to manipulate the host to aid survival at low temperatures, and that aphids may differ in their ability to be manipulated. With recent research suggesting that ectotherms do not possess the physiological thermal safety margin as once thought [55], behavioural thermoregulation could become increasingly integral to the persistence of ectotherms, especially in the face of global climate change. This could put increasing pressure on parasitoids and further increase the fitness benefits to be gained from an ability to manipulate the host to aid thermotolerance.
